# A hyperaccumulation pathway to three-dimensional hierarchical porous nanocomposites for highly robust high-power electrodes

**DOI:** 10.1038/ncomms13432

**Published:** 2016-11-17

**Authors:** Jian Zhu, Yu Shan, Tao Wang, Hongtao Sun, Zipeng Zhao, Lin Mei, Zheng Fan, Zhi Xu, Imran Shakir, Yu Huang, Bingan Lu, Xiangfeng Duan

**Affiliations:** 1 State Key Laboratory for Chemo/Biosensing and Chemometrics, College of Chemistry Hunan University, and School of Physics and Electronics, Changsha 410082, China; 2 Department of Chemistry and Biochemistry, University of California, Los Angeles, California 90095, USA; 3 Institute of Botany, Jiangsu Province and Chinese Academy of Sciences, Nanjing 210014, China; 4 Department of Materials Science and Engineering, University of California, Los Angeles, California 90095, USA; 5 Sustainable Energy Technologies Centre, College of Engineering, King Saud University, Riyadh 11421, Saudi Arabia; 6 California Nanosystems Institute, University of California, Los Angeles, California 90095, USA

## Abstract

Natural plants consist of a hierarchical architecture featuring an intricate network of highly interconnected struts and channels that not only ensure extraordinary structural stability, but also allow efficient transport of nutrients and electrolytes throughout the entire plants. Here we show that a hyperaccumulation effect can allow efficient enrichment of selected metal ions (for example, Sn^2+^, Mn^2+^) in the halophytic plants, which can then be converted into three-dimensional carbon/metal oxide (3DC/MO_*x*_) nanocomposites with both the composition and structure hierarchy. The nanocomposites retain the 3D hierarchical porous network structure, with ultrafine MO_*x*_ nanoparticles uniformly distributed in multi-layers of carbon derived from the cell wall, cytomembrane and tonoplast. It can simultaneously ensure efficient electron and ion transport and help withstand the mechanical stress during the repeated electrochemical cycles, enabling the active material to combine high specific capacities typical of batteries and the cycling stability of supercapacitors.

## Abstract (web summary)

Hyperaccumulation can allow facile enrichment of metal ions in halophytic plants. Here, the authors use the effect to convert plant structures into hierarchical carbon/metal-oxide nanocomposites and demonstrate the structures as battery electrodes combining high power density and excellent cycling stability.

## Graphical abstract

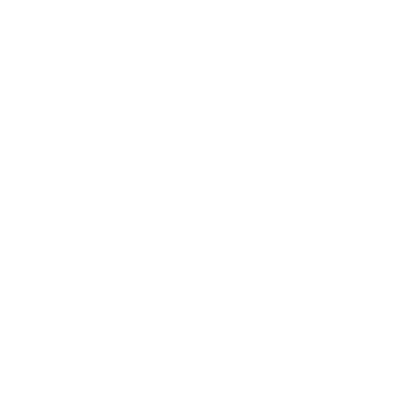

Biomimetic engineering offers a powerful pathway to highly complex material architectures^1^,^2^,^3^,^4^,^5^,^6^ and enable exciting technology opportunities^7^,^8^,^9^,^10^,^11^,^12^,^13^,^14^,^15^,^16^ beyond the reach of conventional synthetic chemistry. Biological structures (for example, woods, bones) typically display a multitude of architectural features with exquisite control of composition and structure hierarchy from nanoscale to macroscopic scale^17^,^18^. The principle of hierarchical design has also been frequently explored for the construction of large manmade structures (for example, Eiffel Tower) and more recently for the creation of ultrastrong, ultralight structural metamaterials^19^,^20^,^21^,^22^. The creation of artificial hierarchical architectures that can mimic natural system with both composition and structure hierarchy has the potential to enable a new generation of materials with tailored microstructures and porosity across multiple length scales and open up totally new technology opportunities in areas ranging from electronics, photonics to energy^7^,^8^,^9^,^10^,^11^,^12^,^13^,^14^,^15^,^16^,^19^,^20^,^21^,^22^. The advancements in synthetic chemistry have led to the production of diverse porous materials with highly ordered pores and exceptional surface area, but typically lacking the structural or compositional hierarchy that is necessary for many essential functions.

Biological structures that result from millions of years of natural evolution and selection have provided many essential materials for human civilization (for example, wood for building construction), and have consistently inspired scientists to design and engineer materials for diverse technologies^3^,^4^,^5^,^6^,^7^,^8^,^9^,^10^,^11^,^12^,^13^,^14^,^15^,^16^. Through photosynthesis, plants convert carbon dioxide, water, other nutrients and minerals from the soil into organic matter, with exquisite hierarchical architectures that is not readily accessible in synthetic materials^23^,^24^,^25^,^26^. Natural plants consist of a hierarchical architecture featuring an intricate network of highly interconnected struts and channels that not only ensure extraordinary structural stability, but also allow efficient transport of nutrients and electrolytes to each individual cell throughout the entire plants. In particular, a class of halophytic plants can grow in soils with very high concentrations of metals salts, absorbing the metal ions through their roots and translocating them into their shoots, and thus concentrating extremely high levels of metals in their tissues. Such selective absorption and enrichment of metal ions through the hyperaccumulation effect has attracted considerable interest for environmental remediation (extracting heavy metals from the contaminated soil) or metal mining^27^,^28^. Alternatively, the hyperaccumulation of metal ions in biological structures could offer an attractive pathway to engineer highly complex composite materials. Various plant structures have been explored for the creation of hierarchical porous carbon structures^29^,^30^,^31^, but not complex composites to date due to rather low mass loading of elements other than carbon in typical plant tissues.

Here we report that a hyperaccumulation effect can be exploited to accumulate a large amount of selected metal ions in halophytic plants, which can then be converted into hierarchically porous three-dimensional (3D) carbon/metal-oxide (3DC/MO_*x*_) nanocomposites with both the composition and structure hierarchy. The resulting 3DC/MO_*x*_ nanocomposites feature a 3D carbon backbone with intertwined microscale struts and nanoscale branches to ensure mechanical strength and facile electron transport; a hierarchical porous structure with highly interconnected micro-channels and nano-channels for highly efficient ion transport throughout the entire network to reach the innermost pores; and uniformly distributed MO_*x*_ nanoparticles in multi-layers of carbon derived from the cell wall, cytomembrane and tonoplast with sufficient internal void spaces to accommodate the volume change and mechanical stress during the repeated electrochemical cycles. The 3DC/MO_*x*_ nanocomposites can thus function as an ideal electrode material for electrochemical energy storage devices. We show a 3DC/SnO_*x*_ nanocomposite derived from *Suaeda glauca* (*S. glauca*) Bunge can function as a highly robust lithium-ion (Li-ion) battery anode with a reversible capacity as high as 802 mAh g^−1^ at a current density of 625 mA g^−1^ for over 3,000 cycles in repeated charge–discharge test over 1 year; and a reversible capacity of 341 mAh g^−1^ over 11,000 cycles at a current density of 12,500 mA g^−1^, an impressive rate capability that is 30–100 times higher than that of the graphite anodes in today's Li-ion batteries.

## Results

### Hyperaccumulation pathway to 3DC/MO_*x*_ nanocomposites

**Figure 1:**
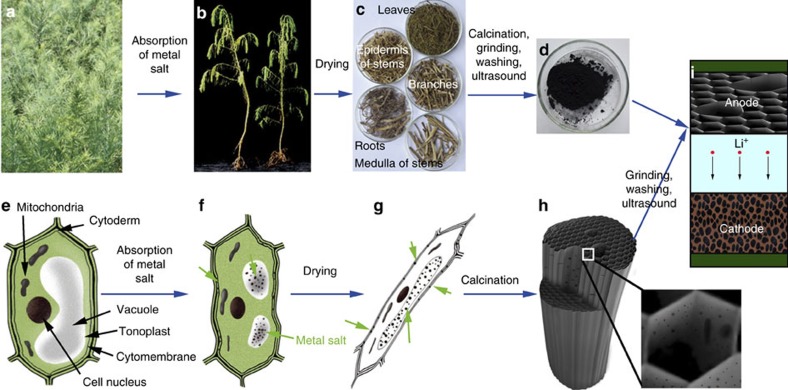
Schematic illustration of three-dimensional carbon/metal-oxide (3DC/MO_*x*_). (**a**,**b**) Photo pictures of the adult plant before (**a**) and after (**b**) cultivating in metal salt solution for 5 days. The *Suaeda glauca* (*S. glauca*) Bunge withered gradually over time due to excessive metal stress. (**c**) Photograph of the dried and separated parts of *S. glauca*, including: roots, epidermis of stems, medulla of stems, branches and leaves. (**d**) Photograph of 3DC/MO_*x*_ after calcination, grinding, washing, ultrasound and drying again. (**e**–**g**) Illustration of morphologic change for the plant cell at different stages of absorption: (**e**) before absorption; (**f**) after absorption for 5 days; (**g**) after drying. (**h**) Schematic illustration of the calcined composite. (**i**) Schematic diagram of the hyperaccumulation produced 3DC/MO_*x*_ as electrode materials for Li-ion batteries.

Figure 1 illustrates the process steps to produce 3DC/MO_*x*_ nanocomposites as battery electrodes from halophytic plants. *S. glauca* is chosen as an example plant because of its exceptional ability to absorb and enrich a large amount of metal ions. The 4-week old *S. glauca* (Fig. 1a) is cultivated hydroponically in ½ MS medium containing 1% selected metal salts (for example, SnCl_2_ for 3DC/SnO_*x*_, or Mn(NO_3_)_2_ for 3DC/MnO) for 5 days to promote the uptake of metal ions. During this process, the metal salts are selectively absorbed by the roots, translocated to and accumulated throughout the entire plant, including the stems, branches and leaves. A series of photographs of the *S. glauca* at different stages of cultivation in metal salts show that the plants withered gradually over time (Fig. 1b; Supplementary Fig. 1) due to excessive metal ion stress. The *S. glauca* was then harvested, separated into roots, epidermis of stems, medulla of stems, branches, and leaves, dried and analysed separately (Fig. 1c). The samples were then annealed at 800 °C for 2 h in an argon atmosphere to produce the 3DC/MO_*x*_ composites (Fig. 1d). Figure 1e–g shows the schematic morphology evolution for the plant cell at different stages of absorption. Figure 1h shows the schematic illustration of the calcined composite. The resulted composite was thoroughly washed to remove the soluble salts (for example, K^+^, Na^+^, Cl^−^ ions) and then used as the anode material for Li-ion batteries (Fig. 1i).

### Hyperaccumulation of metal ions in plant tissue

**Figure 2:**
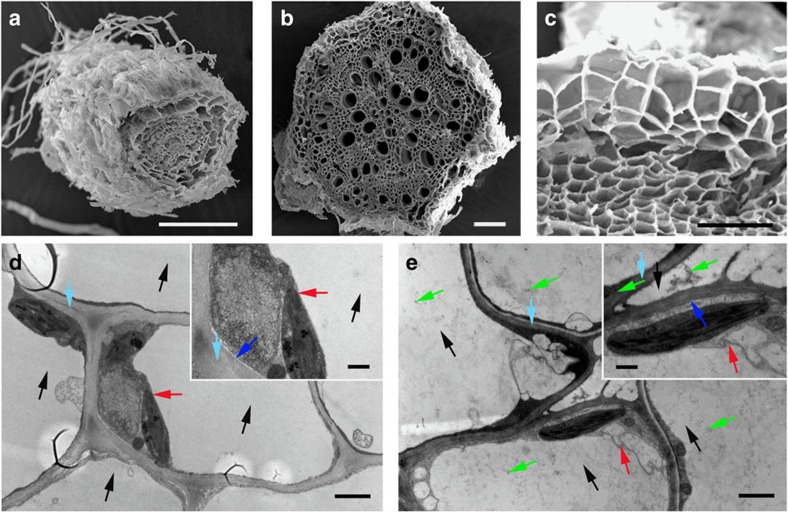
Morphology of the plant cross-sections. (**a**–**c**) Scanning electron microscopy (SEM) images of the plant cross sections after cultivation in 1 wt% tin dichloride solution: (**a**) roots; (**b**) stems; (**c**) epidermis of stems. The three-dimensional hierarchical porous structure of the plant tissue with rich channels and pores across multiple length scales is essential for retaining the structural stability and rapid delivery of electrolyte, ions and nutirents throughout the entire plant to reach each cell. (**d**,**e**) Transmission electron microscopy (TEM) image of the plant cell slice before (**d**) and after (**e**) metal absorption. The black, red, green, blue and cyan arrows represent vacuole, tonoplast, metal salts, cytomembrane and cell wall, respectively. Compared with that before metal absorption, the image of cell slices show rich dark spots in vacuole and cell wall, which corresponds to the accumulated metal salt. The metal salt is distributed in the vacuole with a small amount distributed in the cell wall and cytoplasm. The scale bars in **a**–**e**, inset of d and inset of **e** are 200, 200, 50, 2, 2, 0.5 and 0.5 μm, respectively.

Scanning electron microscopy (SEM) images of the roots (Fig. 2a), stems (Fig. 2b) and epidermis of the stems (Fig. 2c) clearly show hierarchical porous structures containing abundant channels of varying sizes, which is highly favourable for efficient absorption and transport of nutrients and metal ions from the roots to the shoots. Elemental analysis of the *S. glauca* by energy dispersive X-ray spectroscopy (EDS) indicates that a variety of elements are present including C, O, Na, Mg, Al, Si, K, Ca, Fe and Sn (Supplementary Fig. 2). In particular, comparing to *S. glauca* without hyperaccumulation process in which Sn is essentially not detectable, the mass fraction of Sn in epidermis of the stems is significantly increased to reach up to ∼20% of total mass after drying, indicating highly effective extraction and enrichment of the selected metal ions from the cultivation solution. It is interesting to note that the mass fractions of K, Na ions, and so on decrease after absorption of Sn (Supplementary Fig. 2). Since Sn has no exclusive ion channel in *S. glauca*, it could occupy other ion channels^32^. Under condition of the high chemical potential gradient of tin salt, non-selective ion channels can absorb tin ions to facilitate diffusion and intake of Sn, resulting in a large amount of Sn in *S. glauca* along with decreased K, Na concentration.

Transmission electron microscopy (TEM) images of cell slice for epidermis of stems before (Fig. 2d) and after (Fig. 2e) hyperaccumulation process clearly shows vacuole, cell nucleus, mitochondria, chloroplast, cytoplasm and cell wall. It is apparent that the absorbed metal ions are primarily enriched in the vacuole, with a small amount also absorbed on the cell wall, cytomembrane and cytoplasm (Fig. 2e; Supplementary Fig. 3). It is known that excessive metal salts can harm the typical plant growth^33^. Cell wall, an ion exchanger of relatively low affinity and low selectivity, is the first barrier for metal salts to enter the cytoplasm, which blocks the metals reaching into the interior of cell by adsorption, complexation and sedimentation^34^, leading to the immobilization of metal salts in the cell wall (Supplementary Fig. 3). After the quantity of metal salt in cell wall reaches the maximum level, the remaining metal ions can transfer to the plasmogen through cytomembrane. The membrane potential, which is negative on the inside of the plasma membrane, provides a strong driving force for the intake of metal salts through secondary transporters^35^. Then majority metal salts are sequestered and accumulated in cell vacuoles. Vacuole has abundant organic acid, protein and alkaloid that can coordinate with metal ions to form complexes and decrease their impact on cell activity^36^.

It is interesting to note that the epidermis of the stems contains the greatest amount of Sn (see Supplementary Fig. 2: 21.6% for epidermis of stems, 13.9% for roots and 7.1% for medulla of stems). The additional enrichment of Sn in the epidermis agrees well with the self-protection mechanism of *S. glauca*^37^,^38^. Complex interactions of transport and chelating activities control the rates of metal ion intake and accumulation in different parts of the plant. Normally, the metal salt tolerance and detoxification mechanism include external rejection and interior tolerance^34^. For *S. glauca*, metal salts can accumulate in the sensitive site inside the cells (for example, vacuole). Meanwhile, the cells pump out the excessive metal salts, transfer them into intercellular space, and accumulate in epidermis passing through cortex^37^,^38^. In this way, more and more tin salts were accumulated in epidermis over time, leading to a highest content of Sn in epidermis of stems.

### Structural hierarchy in 3DC/MO_*x*_ nanocomposites

**Figure 3:**
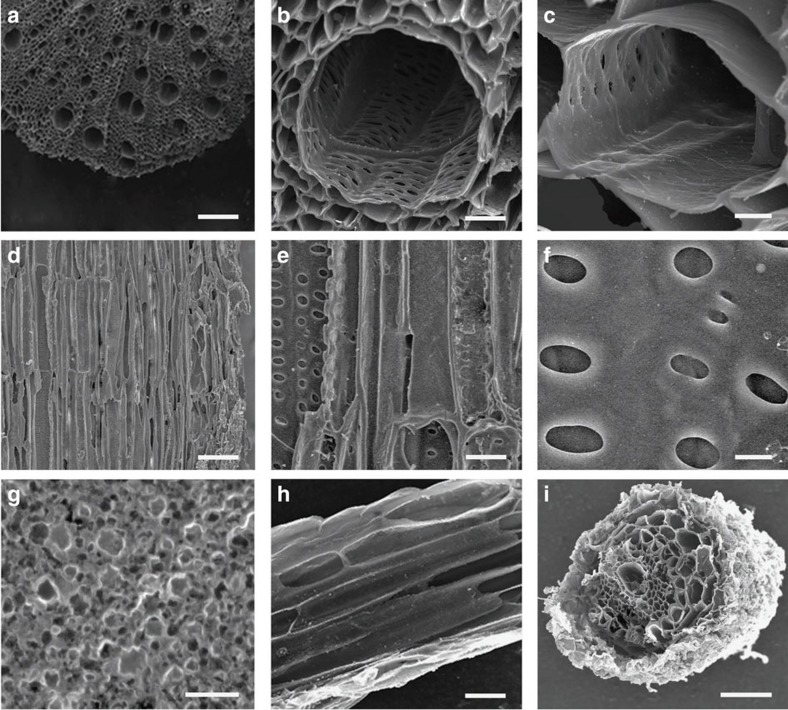
Morphology of three-dimensional hierarchical porous composites. (**a**–**c**) SEM images of cross section of calcined composite derived from medulla of stems show unique 3D hierarchical porous structure. The parallel channels derived from vasculature with the diameters on the order of 50 μm are surrounded by the parenchymal cells with abundant pores across the cell wall. (**d**–**f**) SEM images of the longitudinal section of stems at different magnifications, clearly show the larger channels of the vasculature and elongated parenchymal cells. (**g**) A further closeup view displays a typical mesoporous structure with abundant sub-10 nm pores. (**h**) Longitudinal section of calcined composite derived from epidermis of stems. (**i**) Cross-section of calcined composite derived from the roots. The microscopy image studies show clearly that the hierarchical porous structure of the plant tissue is well retained after the carbonization process, to produce a calcined composite with interpenetrating networks of carbon backbone for efficient electron transport, and porous channels for rapid ion transport. The scale bars in **a**–**i** are 100 μm, 10 μm, 2 μm, 100 μm, 10 μm, 2 μm, 30 nm, 10 μm and 60 μm, respectively.

The harvested and separated parts of *S. glauca* were next dried and calcined in argon atmosphere at 800 °C for 2 h to obtain the calcined composite. Figure 3 shows a series SEM images of the resulted composite derived from different parts of *S. glauca*. It is evident the 3D hierarchical porosity is well retained in all calcined samples (Fig. 3; Supplementary Fig. 4). Taking the samples derived from the stems as an example, the SEM images of cross-section show that the calcined stem medulla displays a high density array of parallel channels corresponding to larger catheter with the diameters on the order of 50 μm surrounded by the parenchymal cells with diameters on the order of 5 μm (Fig. 3a–c). The high-resolution SEM images also clearly show that the sidewalls of these channels exhibit abundant transverse pores with pore sizes in the range of 1–5 μm (Fig. 3b,c). The SEM images of longitudinal section show similar structures with long parallel channels along the longitudinal direction that correspond to larger vasculature and elongated parenchymal cells (Fig. 3d–f). The high resolution SEM image of the side wall further confirms the existence of abundant transverse pores on the order of a few micrometers derived from pits of the plant (Fig. 3f). A further closeup view of the calcined cell wall structure reveals a high density of ultrafine pores with the size on the order of 1–10 nm (Fig. 3g), which are derived from plasmodesmata (that is, microscopic channels traversing the cell walls of plant cells). SEM studies of the stem epidermis and the roots show highly comparable structures (Fig. 3h,i; Supplementary Fig. 4). Together, these studies clearly demonstrate that the hierarchical porous structures of *S. glauca* are well retained during the calcination process.

Plants catheter is a relatively perfect structure for water transportation. Aqueous nutrients flow smoothly through the catheter cell cavity to rise up along the stem, along with the transverse transportation through the pores on the sidewalls to the surrounding parenchyma cells. Together, these channels and pores with sizes ranging from a few tens of micrometers down to a few nanometers forms a well-integrated hierarchical porosity with a fully interconnected continuous network of channels that is ideally suited for highly efficient mass transport, with the larger channels responsible for the rapid transport of the electrolytes, to the entire structures, and smaller ultrafine channels and pores facilitating the effective delivery of the ions and nutrients into each cell. The preservation of the hierarchical porosity in the resulting calcined composite can simultaneously offer highly effective electron conductive pathways through the carbon backbones and highly efficient ion transport pathways through the interconnecting micro- and nano-channels and pores.

### Compositional hierarchy in 3DC/MO_*x*_ nanocomposites

**Figure 4:**
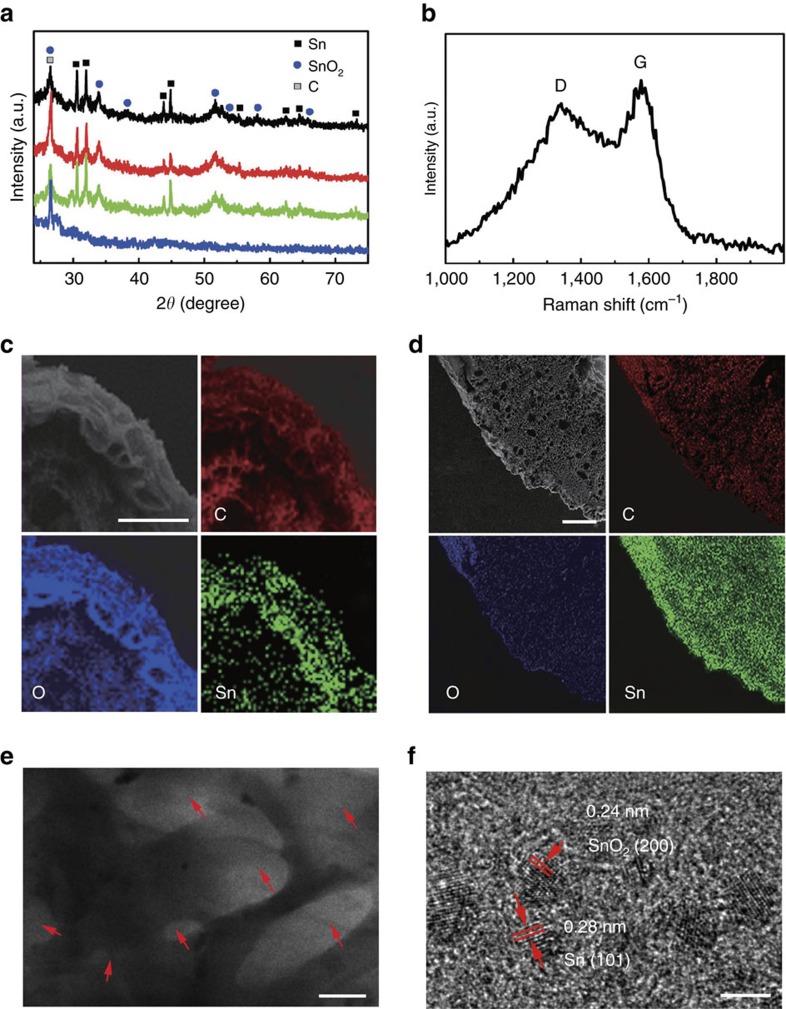
Morphology and structure characterization of 3DC/SnO_*x*_ hierarchical porous composites. (**a**) X-ray diffraction pattern of the samples after washing derived from roots (3DC/SnO_*x*_-R, black), epidermis of stems (3DC/SnO_*x*_-E, red), medulla of stems (3DC/SnO_*x*_-M, green) and pure 3D carbon derived from epidermis of stems (3DC-E, blue). Most of the peaks agree well with the metallic Sn and the rutile phase SnO_2_. The diffraction peak 26.4° consists of a relatively broad base and sharp peak, which can be attributed to the overlap of SnO_2_ and graphite diffraction. (**b**) Raman spectra of the 3DC/SnO_*x*_-E show clear D and G band with a relatively small D/G-band peak intensity ratios, indicating the 3DC/SnO_*x*_-E is partially graphitized and well ordered. (**c**) SEM and energy dispersive X-ray spectroscopy (EDS) elemental mapping of cross section of the 3DC/SnO_*x*_-R. (**d**) SEM and EDS elemental mapping of the cross-section of the stems reveals the epidermis show slightly higher tin content. (**e**) TEM image of the 3DC/SnO_*x*_-E with abundant void space. The red arrows highlight the pores derived from the parenchymal cells. (**f**) High-resolution TEM image of the 3DC/SnO_*x*_-E shows the ultrafine SnO_*x*_ nanoparticles are well embedded in the carbon matrix. The scale bars in **c**,**d**,**e** and **f** are 50 μm, 200 μm, 1 μm and 5 nm, respectively.

We have next used X-ray diffraction to evaluate the resulted 3DC/MO_*x*_ composites. X-ray diffraction pattern of the as-prepared 3DC/SnO_*x*_ show that most of the peaks can be readily indexed to metallic Sn (JCPDS No.04-0673) and the rutile phase SnO_2_ (JCPDS No. 41–1445) (Fig. 4a). Similarly, the X-ray diffraction pattern of the as-prepared 3DC/MnO shows that most of the peaks can be readily indexed to MnO (JCPDS No. 07-0230) (Supplementary Fig. 5). It is important to note that the diffraction peak at 2*θ*=26.4° (d-spacing: 0.34 nm) consists of a relatively broad base and sharp peak, which can be attributed to the overlap of SnO_2_ (110) lattice spacing and the inter-layer distance of graphite, suggesting that the carbon in the 3DC are partially graphitized and well ordered. The formation of graphitic carbon is also confirmed in pure 3DC (blue curve in Fig. 4a) and 3DC/MnO composite (Supplementary Fig. 5) where a clean diffraction peak at 2*θ*=26.4° is also seen without interference from SnO_2_. Raman spectroscopy studies also reveal a prominent G band for the 3DC/MO_*x*_ calcined at 800 °C (Fig. 4b). The small D/G-band peak intensity ratios of 3DC/MO_*x*_ confirm the formation of graphitized carbon, which is important for ensuring high electron conductivity of the electrode materials^39^.

Elemental analysis by inductively coupled plasma atomic emission spectroscopy (ICP-AES) demonstrates that the 3DC/SnO_*x*_ composites contain ∼32%, 41% and 17% of Sn in mass for the composites derived from the roots (3DC/SnO_*x*_-R), epidermis of stems (3DC/SnO_*x*_-E) and medulla of stems (3DC/SnO_*x*_-M), respectively. The highest Sn content in the epidermis agrees well with elemental analysis before calcination. EDS studies of 3DC/SnO_*x*_-E further confirm a high content of Sn (40.0%), carbon (47.1%), oxygen (9.7%) in the composite, with other impurities (for example, Si, Na, K, Ca, Mg, Fe, N) around 1% or less (Supplementary Table 1). We have further used EDS mapping to evaluate the spatial distribution of metallic elements across the entire structure. The EDS mapping images of the cross-section of 3DC/SnO_*x*_ derived from the *S. glauca* roots and stems clearly shows that Sn is well distributed throughout the entire structure (Fig. 4c,d). There is apparently more enrichment of Sn towards the epidermis of stems, which is consistent with our element analysis of the separated parts. The morphology and structure of the 3DC/SnO_*x*_ were also investigated by TEM. TEM image clearly shows porous structure of the 3DC backbone (Fig. 4e) and HRTEM image show abundant ultrafine Sn or SnO_2_ nanoparticles well embedded in the carbon matrix (Fig. 4f). The Brunauer–Emmett–Teller (BET) analysis further demonstrates that the 3DC/SnO_*x*_ composites exhibit a high specific surface area up to 484 m^2^ g^−1^ with a large population of sub-10 nm pores (Supplementary Fig. 6).

The above studies clearly show that the hyperaccumulation effect can be utilized for creating highly uniform 3D composite materials with well-integrated hierarchical porosity. The 3D hierarchical network with high electronic conductivity and structural stability could provide an excellent conductive scaffold for enhanced performance in electrochemical energy storage devices^40^. The resulting 3DC/MO_*x*_ composite exhibits several unique features to make it an ideal electrode material for highly robust electrochemical energy storage devices. First, the 3D carbon backbone features well-joined larger struts with fine branches to ensure mechanical strength and electron transport; second, the hierarchical porous structure consists of highly interconnected micro-channels and nano-channels for highly efficient ion transport across the entire network; third, the uniform-distribution of MO_*x*_ nanoparticles within the high surface area 3DC matrix can ensure efficient electron transfer to/from the 3DC and ion transport to/from the electrolyte; and lastly, the multi-layers of carbon derived from the cell wall, cytomembrane and tonoplast with sufficient internal void spaces can accommodate the volume change and mechanical stress of MO_*x*_ nanoparticles during electrochemical cycles.

### Electrochemical evaluation of the 3DC/SnO_*x*_ anodes

The hierarchical porous 3DC/MO_*x*_ nanocomposite can simultaneously boost electron and ion transport and withstand the mechanical stress during the repeated electrochemical cycles. To evaluate the electrochemical performance of the resulted composites, we have explored the 3DC/SnO_*x*_ as the anode material for lithium ion batteries. A number of metal oxides including SnO_*x*_ has attracted considerable interest as a potential anode material for Li-ion batteries for their large specific capacity, but typically with limited cycling stability (particularly under high current densities) due to large volume expansion induced strain and disintegration of electrode material during the repeated charge/discharge cycles^39^,^41^,^42^,^43^,^44^,^45^,^46^,^47^,^48^,^49^,^50^,^51^,^52^,^53^,^54^. The cyclic voltammogram of 3DC/SnO_*x*_-E versus Li^+^/Li and charge–discharge profiles of 3DC/SnO_*x*_ composite electrodes (Supplementary Fig. 7) show typical electrochemical characteristics of SnO_*x*_ based electrodes^41^,^42^,^45^,^47^,^48^,^49^,^52^.

**Figure 5:**
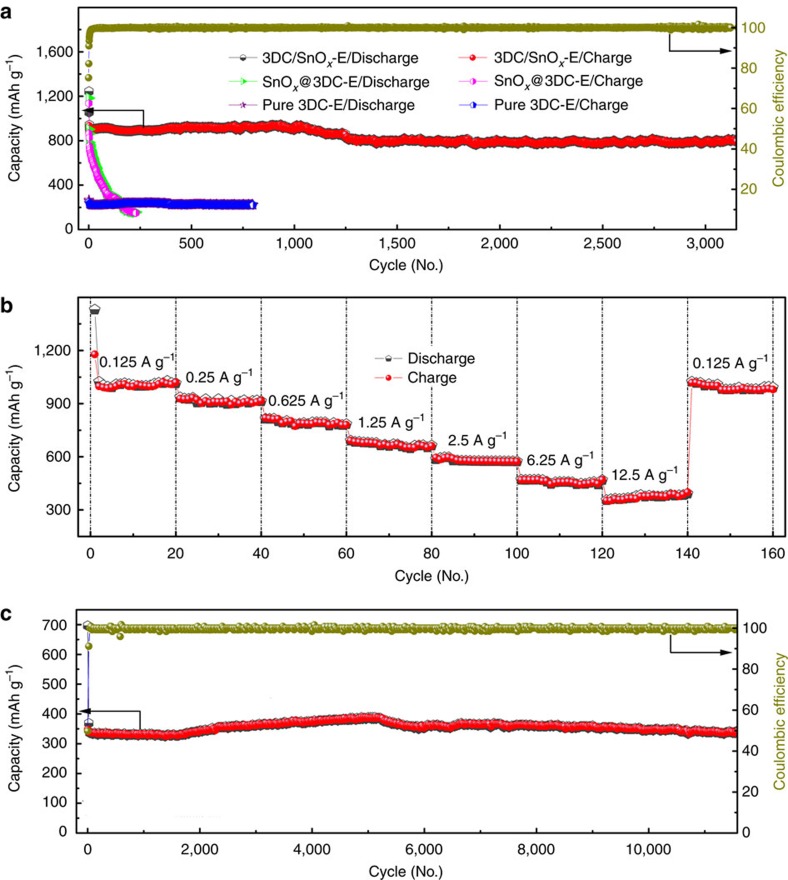
Electrochemical properties of the 3DC/SnO_*x*_ anode. (**a**) Cycle performance of the anodes made from hyperaccumulation derived 3DC/SnO_*x*_-E, pure 3D carbon derived from epidermis of stems with chemically/physically loaded SnO_*x*_ (SnO_*x*_@3DC-E), pure 3DC-E at a current density of 625 mA g^−1^. The 3DC/SnO_*x*_-E anode shows a highly robust performance, delivering a reversible capacity of 802 mAh g^−1^ over 3,000 repeated charge/discharge cycles during 1-year of continuous testing, with a Coulombic efficiency >99% after initial 5th cycles (dark yellow line). In contrast, the pure 3DC-E electrode shows a stable but relative low reversible capacity of 278 mAh g^−1^ and the chemical derived SnO_*x*_@3DC-E mix show a comparable initial capacity that quickly degrade to <160 mAh g^−1^ within about 230 cycles. These studies clearly highlight that the hyperacummulation process is essential for creating highly robust composite electrode. (**b**) Rate performances of the 3DC/SnO_*x*_-E at different current density. All specific capacities of 3DC/SnO_*x*_ anodes are based on the total mass of 3DC/SnO_*x*_. The 3DC/SnO_*x*_-E anodes delivers a reversible capacity of 392 mAh g^−1^ at a current density of 12,500 mA g^−1^, an excellent rate capability that is 30–100 times higher than that of the graphite anodes in today's Li-ion batteries. (**c**) Cycling performance of 3DC/SnO_*x*_-E at the high current density of 12,500 mA g^−1^, demonstrating excellent cycling stability even at ultra-high current density. The dark-yellow line shows the Coulombic efficiency.

Significantly, the charge/discharge cycling behaviour of our 3DC/SnO_*x*_-E anode shows a highly robust performance during more than 1 year of continuous testing at a current density of 625 mA g^−1^, displaying a reversible capacity of 802 mAh g^−1^ for over 3,000 repeated charge/discharge cycles (Fig. 5a). The Coulombic efficiency for our 3DC/SnO_*x*_-E anode was more than 99% after the initial 5 cycles, demonstrating an excellent cycling stability (0.01% decay per cycle), representing, to our knowledge the best cycling stability ever achieved in SnO_*x*_ anodes reported to date. It is worth noting that the capacitance experienced an increase, fading, and then another slight increase, which could be partly attributed to local temperature swing (Changsha, China) during the long testing period (>1 year). For comparison, we have also prepared two control samples, by carbonizing the epidermis of stems without hyperaccumulation process to obtain 3D carbon without SnO_*x*_ (3DC-E) as the first control sample, and by mixing 3DC-E with tin (II) chloride dihydrate followed by a similar calcination process (see Methods) to obtain 3DC-E physically/chemically loading SnO_*x*_ (SnO_*x*_@3DC-E) as the second control sample. Our control experiment with the 3DC-E electrode shows a highly stable but relatively low reversible capacity of 278 mAh g^−1^ after 800 cycles, comparable with typical graphite electrode. The SnO_*x*_@3DC-E electrode shows a comparable initial capacity to that of hyperaccumulation prepared 3DC/SnO_*x*_-E electrode, but quickly degrades to <160 mAh g^−1^ in about 230 cycles. These studies clearly highlight that the hyperaccumulation process is essential for creating highly robust composite electrodes.

Rate performance (or specific power) is an important figure of merit for battery operation, particularly for practical applications in electric vehicles (EVs) or power tools that require high power^55^,^56^. The achievement of high power in batteries has been a challenge due to the relatively slow ion and/or electron transport in typical electrode design today. With an interpenetrating network of carbon backbones and hierarchical porous channels, the 3DC/SnO_*x*_ not only simultaneously offers electron transport and ion transport pathways, but also ensures highly efficient electrochemical reaction in the well-distributed SnO_*x*_ nanoparticles to promise exceptional rate performance. Indeed, the rate performance test of 3DC/SnO_*x*_-E anode at variable current density shows impressive rate capability in 3DC/SnO_*x*_ with a reversible capacity of 1014, 914, 784, 663, 574, 468 and 392 mAh g^−1^ at the current densities of 125, 250, 625, 1,250, 2,500, 6,250 and 12,500 mA g^−1^, respectively (Fig. 5b). The reversible capacity of 1,014 mAh g^−1^ achieved at 125 mA g^−1^ is about three times of that of the graphite anode achieved at a similar current density. We note that the achieved capacity exceeds the theoretical capacity of the composites, which has also been observed in synthetic carbon/SnO_*x*_ composites previously and can be attributed the synergistic effect of hierarchical nanocomposites^39^. Cyclic voltammogram studies at variable scan rates demonstrate that our 3DC/SnO_*x*_-E anode clearly exhibits faster mass transport kinetics than the SnO_*x*_@3DC-E control electrode (Supplementary Fig. 8), which can be attributed to the favourable ion transport kinetics through the hierarchical porous structure and interfacial storage (that is, pseudo-capacitance-type behaviour) provided by interface sites of nano-sized SnO_*x*_ (refs 57, 58). It is particularly noteworthy such a high capacity can be achieved in the 3DC/SnO_*x*_ with relatively low mass loading of SnO_*x*_ (∼41% Sn) suggesting that the overall capacity may be further improved if the mass loading can be improved in future studies. It is also noteworthy that a reversible capacity (392 mAh g^−1^) comparable to that of traditional graphite anode can be achieved at the very high current density 12,500 mA g^−1^, which is about 30–100 times higher than the typical charge/discharge current density used in today's Li-ion batteries. Such a high current density compares well with those of current supercapacitors.

With the hierarchical carbon network and well-encapsulated SnO_*x*_ to withstand the repeated mechanical stress during the charge/discharge cycles, the 3DC/SnO_*x*_ can deliver impressive cycling robustness even under high charge/discharge current density. The 3DC/SnO_*x*_-E delivers highly reversible capacities of 551 mAh g^−1^ after 6,000 cycles at the current density of 2,500 mA g^−1^ (Supplementary Fig. 9a), 380 mAh g^−1^ after 10,000 cycles at the current density of 6,250 mA g^−1^ (Supplementary Fig. 9b) and 341 mAh g^−1^ after 11,000 cycles at the current density of 12,500 mA g^−1^ (Fig. 5c). To our knowledge, such cycling stability has not been achieved in SnO_*x*_-based electrodes previously and is generally difficult to achieve in most battery electrodes particularly at such high charge/discharge current densities, and is typically only achieved in supercapacitors.

We have also conducted similar studies and achieved the reversible capacity of 663 mAh g^−1^ for 3DC/SnO_*x*_-R and 601 mAh g^−1^ for 3DC/SnO_*x*_-M at 2,000th cycle at the current density of 625 mA g^−1^ along with comparable rate performance (Supplementary Fig. 10). The slightly lower capacity than that in 3DC/SnO_*x*_-E can be largely attributed to lower SnO_*x*_ mass ratio in these materials (The capacity is normalized by the total mass of the 3DC/SnO_*x*_). These studies clearly demonstrate that 3DC/SnO_*x*_ can function as a Li-ion anode material with excellent cycling robustness and rate capability. Additionally, similar results were also obtained from the 3DC/MnO derived from *S. glauca* (Supplementary Fig. 11).

## Discussion

The electrochemical performance demonstrated in the 3DC/MO_*x*_ can be attributed to its unique morphology and hierarchical architecture of the hyperaccumulation derived nanocomposites. Within the 3DC/MO_*x*_, ultrafine MO_*x*_ nanoparticles are uniformly distributed within the 3D hierarchically porous carbon matrix. First, most of the nanoparticles were well-distributed in the triple carbon layer derived from cell wall, cytomembrane and tonoplast. A stable solid electrolyte interface (SEI) formed on the outer carbon layers, minimizing the amount of SEI and ensuring its stability. High resolution TEM image of the 3DC/SnO_*x*_-E reveals the SEI (∼10–25 nm) on the surface of the carbon matrix is highly stable over the long lifetime cycling testing period (Supplementary Fig. 12a,b). Second, the hierarchical porous structure offers sufficient internal void space, which can accommodate large volume change in MO_*x*_ nanoparticles to withstand the mechanical stress and ensure exceptional cycling stability during the repeated electrochemical cycles. TEM and SEM studies of the resulted 3DC/SnO_*x*_-E composite show that the hierarchical porous structure of the composite electrode is largely intact over the cycling period (Supplementary Fig. 12c–f), which can facilitate lithium transport kinetics and deliver sustained capacity and robust cycling performance. Third, the well-distributed ultrafine MO_*x*_ nanoparticles in 3D carbon matrix can allow rapid electrochemistry and readily accommodate large (de)lithiation strain without fracture^43^,^59^,^60^,^61^. Fourth, the hierarchical high surface area porous structures across multiple length scales (including macropore, mesopore and micropore) offer the most efficient pathway for rapid transport and deliver of Li^+^ throughout the entire 3D architecture, to the innermost region of the electrode.

In conclusion, we have demonstrated that basic botanic principles can be readily applied for the rational design of microscopic 3D architectures with both compositional and structural hierarchy. We have shown that the hyperaccumulation effect can be exploited to enrich selected metal species in plant tissues, which can then be converted to highly complex composite materials with an precise control of the chemical composition and architecture hierarchy. We show the hyperaccumulation derived 3DC/SnO_*x*_ nanocomposites exhibit a hierarchical porous structure that can simultaneously aid electron and ion transport and help withstand the mechanical stress during the repeated electrochemical cycles, and function as a battery electrode material combination of high power density, high active material utilization and superior cycling endurance. This strategy may be extended to other halophytic plants or metals for the creation of a wide array of composite materials with tailored chemical composition, physical architecture, and electronic, optical or magnetic functions. It establishes a possible new avenue for engineering complex materials for diverse technologies including supercapacitors, batteries, sensing, catalysis and fuel cells.

## Methods

### Chemicals

Tin dichloride dihydrate (SnCl_2_·2H_2_O, >99.99%), manganese nitrate tetrahydrate (Mn(NO_3_)_2_·4H_2_O, >99.99%) were purchased from Sigma-Aldrich. All the chemicals were used as received without further purification. The water (18 MΩ cm^−1^) used in all experiments was prepared by passing through an ultra-pure purification system (Aqua Solutions).

### Synthesis of 3DC/MO_*x*_

The naturally growth seedling was transplanted and grown hydroponically in aqueous solution with ½ MS medium for 4 weeks to obtain 20–50 cm tall *S. glauca*, and then in ½ MS medium with 1% selected metal salts (for example, SnCl_2_ for 3DC/SnO_*x*_, or Mn(NO_3_)_2_ for 3DC/MnO) for 5 days for hyperaccumulation of metal ion in the plants. After that, the *S. glauca* were thoroughly washed and dried, and separated into the roots (root bark and root pulp), epidermis of stems (obtained from lower part of stems, including part of cortex), medulla of stems (obtained from lower part of stems), branches and leaves, and calcined at 800 °C for 2 h in argon atmosphere at a ramping rate of 2 °C min^−1^. The resulted samples were ground. One gram of 3DC/MO_*x*_ was added into 50 ml deionized water followed by stirring and ultrasound for 20 min, respectively. The products were separated by centrifugation, washed three times at room temperature. Similarly, the samples were dispersed in ethanol and washed three times. The resulting samples were also separated by centrifugation, and dried in an oven at 80 °C for 24 h. As a control, a pure 3DC-E sample was obtained from epidermis of stems of *S. glauca* without the hyperaccumulation process. The resulted 3DC-E and tin (II) chloride dihydrate with a mass ratio about 1.5 were added into 20 ml deionized water, stirring, dried and annealed at 800 °C for 2 h in argon to obtain physically/chemically derived SnO_*x*_@3DC-E composite.

### Material characterizations

Morphology characterizations were performed by using SEM (Hitachi S-4800, 5 kV) as well as EDS microanalysis. A JEOL JEM-2100F microscope operated at 200 kV in a scanning mode with a nominal analytical beam size of 0.5 nm was used to conduct the TEM, high-resolution TEM investigations. Crystal structures of samples were characterized with power X-ray diffraction (Philips, X'pert pro, Cu Ka, 0.154056, nm). The X-ray diffraction specimens were prepared by means of grinding and flattening the powders on small slides. ICP-AES (Shimadzu ICPE-9000) was used to determine the mass fraction of Sn in 3DC/MO_*x*_. Raman spectroscopic analysis was conducted with an RM3000Micro Raman System (Renishaw PLC) with argon laser excitation at 532 nm. Specific surface areas were measured by using BET nitrogen adsorption-desorption (NOVA 2200e, Quanthachrome, USA.) at 77 K and pore size distributions were calculated from the adsorption branch of the Nitrogen adsorption–desorption isotherm via the Barrett Joyner Halenda formula.

### Electrochemical measurements

The electrochemical properties of the samples were studied using CR2025-type coin cells. The composites derived from the roots, epidermis of stems, medulla of stems of hyperaccumulated *S. glauca*, as well as the control samples 3DC-E and SnO_*x*_@3DC-E were used as the anode materials with the separator of Separion S240 P25 (Degussa). The loading mass of active materials on electrode ranges from 0.35–0.88 mg cm^−2^. 1M LiPF_6_ in ethylene carbonate and diethyl carbonate (1:1 v/v) was used as electrolyte. The volume of electrolyte is 0.05 ml cm^−2^. Lithium ribbon (Aldrich) was used as the counter electrode. The active materials, conductive carbon black, and polyvinylidene fluoride in the weight ratio 80:10:10 were combined to prepare the working electrodes. The slurry was cast onto copper foil by using the doctor-blade technique and then dried under vacuum at 80 °C for 12 h before assembly. Standard half-cells were fabricated inside a glove box with water and oxygen contents of <0.5 p.p.m. Galvanostatic charge-discharge cycles were carried out by a computer controlled battery tester system (Arbin BT-2000) at a current density of 100, 200, 500, 1,000, 2,000, 5,000 and 10,000 mA g^−1^ in the voltage range of 0.01–3.0 V. Cyclic voltammogram curves were measured using an electrochemical workstation from 3.0 to 0.01 V (versus Li^+^/Li).

### Data availability

The authors declare that the data supporting the findings of this study are available within the paper and its Supplementary Information files.

## Additional information

**How to cite this article:** Zhu, J. *et al*. A hyperaccumulation pathway to three-dimensional hierarchical porous nanocomposites for highly robust high-power electrodes. *Nat. Commun.* **7,** 13432 doi: 10.1038/ncomms13432 (2016).

**Publisher's note:** Springer Nature remains neutral with regard to jurisdictional claims in published maps and institutional affiliations.

## Supplementary Material

Supplementary Information Supplementary Figures 1-12, Supplementary Table 1 and Supplementary References
