# Supplementary material for: A hyperaccumulation pathway to three-dimensional hierarchical porous nanocomposites for highly robust high-power electrodes
Source: Nat Commun. 2016 Nov 17;7:13432. doi: 10.1038/ncomms13432 (PMC5118540; doi:10.1038/ncomms13432)
Supplement: Supplementary Information — Supplementary Figures 1-12, Supplementary Table 1 and Supplementary References [file ncomms13432-s1.pdf]

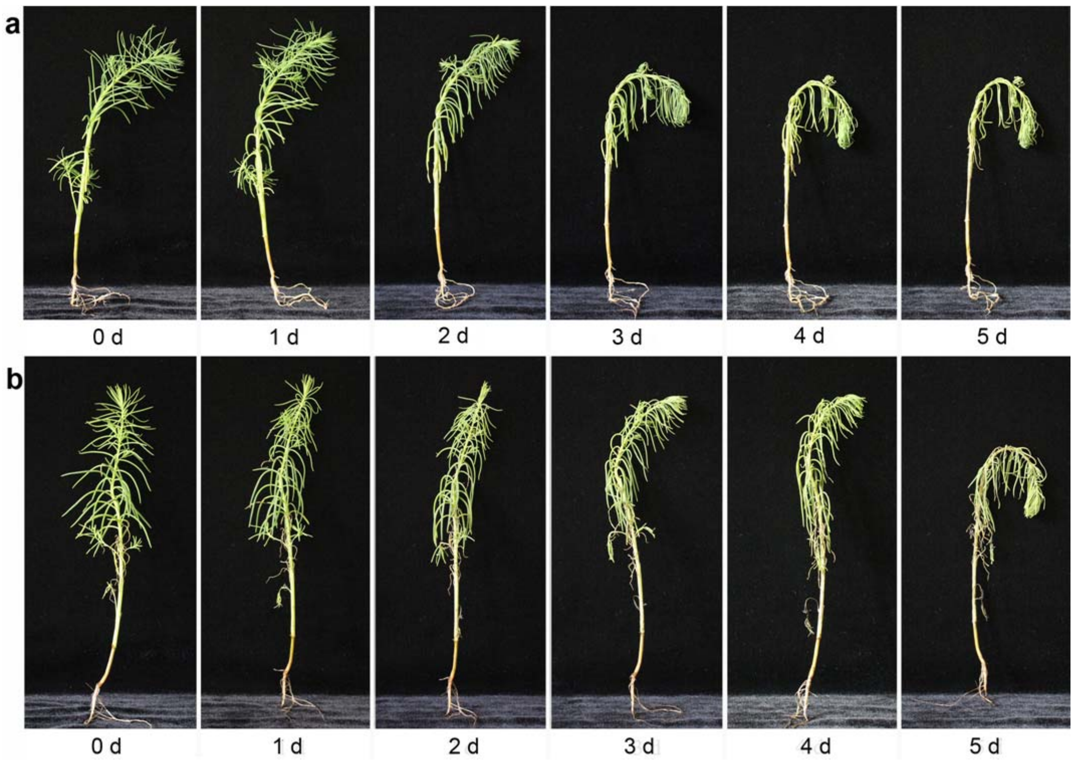

Supplementary Figure 1. Photographs of the *Suaeda glauca* (*S. glauca*) Bunge at different stages of metal ion absorption. (a) Photographs of *S. glauca* after absorption of tin salt. (b) Photographs of *S. glauca* after absorption of manganese salt. It is apparent that the plants withered over time under high concentration metal stress. It is noteworthy is that the halophytic *S. glauca* did not completely wither after 5 days, allowing sufficient time to accumulate a larger amount of metal salt.

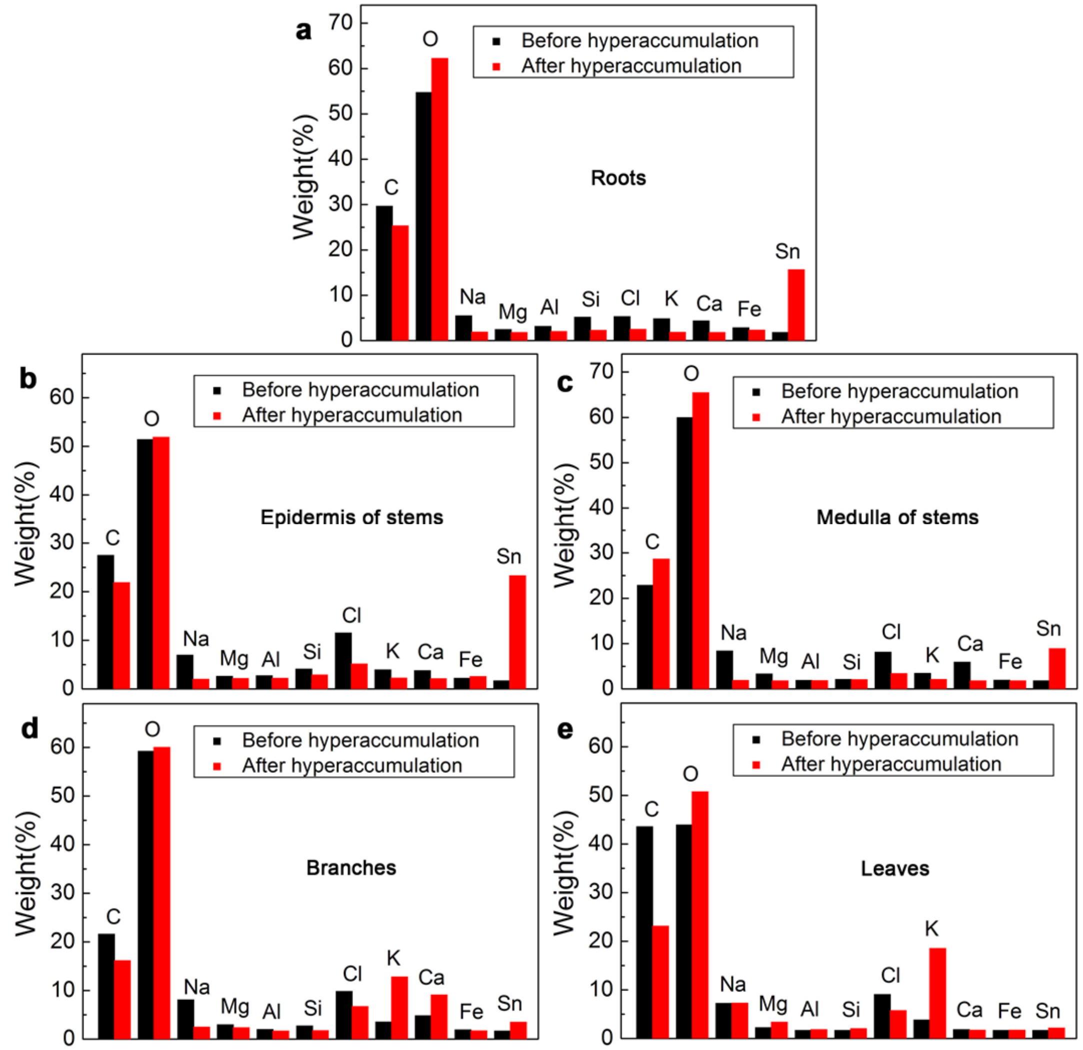

Supplementary Figure 2. Elemental composition of the different parts of plant slice with and without hyperaccumulation after drying. (a) Roots; (b) Epidermis of stems; (c) Medulla of stems; (d) Branches; (e) Leaves. It is evident that there are considerable amount of Sn accumulated in roots (13.86%), epidermis of stems (21.61%) and medulla of stems (7.12%). Once Sn is adsorbed on surface of roots, it can be transferred to the inside of the roots through apoplast and symplasm[1]. Roots plasma membrane,vacuole, etc. are also important site of Sn accumulation[2]. So the roots have a large content of Sn. In general, metal sals have to be absorbed into the root symplasm before they can enter the xylem, because the casparian strip act as a barrier for apoplastic diffusion into the medulla[3]. The adsorbed metal salts transferred to the aboveground part is controlled by two processes: transferring from parenchymal cell in xylem to vessel and transferring within the vessel[4]. As we know, 1 wt% tin dichloride dehydrate exceeds the *S. glauca* tolerance limitation (see Supplementary Fig. 1). *S. glauca* couldn’t sustain normal physiological function above 5 days in 1% tin dichloride aqueous solution. So *S. glauca* has not enough time to adsorb and accumulate a lot of Sn in its branches and leaves. So the branches and leaves have smaller content of Sn.

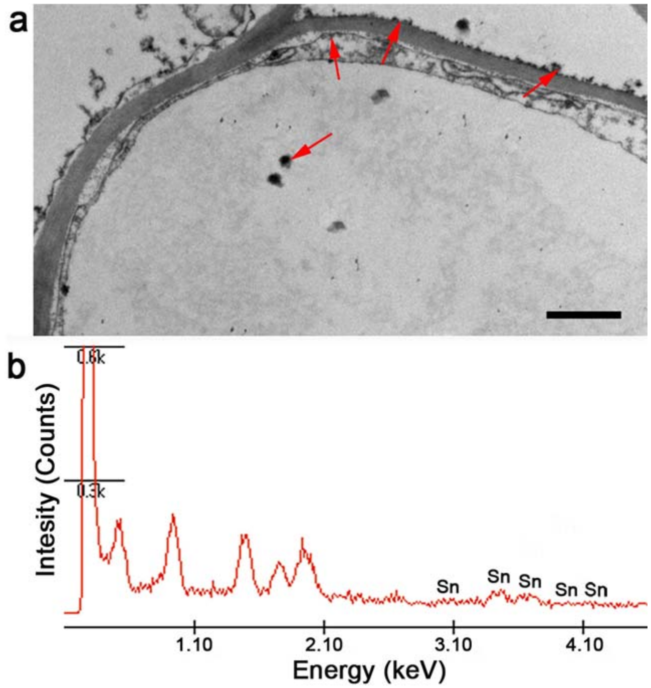

Supplementary Figure 3. Transmission electron microscopy (TEM) and (energy dispersive X-ray spectroscopy) EDS of plant slice after hyperaccumulation process. (a) TEM of plant cell slice prepared from the *S. glauca* after hyperaccumulation process. (b) TEM Energy Dispersive Spectrum (TEM-EDS) of plant cell slice prepared from the *S. glauca* after hyperaccumulation process, which shows the Sn distributes in vacuole, cell wall and cytoplasm. The scale bar in a is 1 μm.

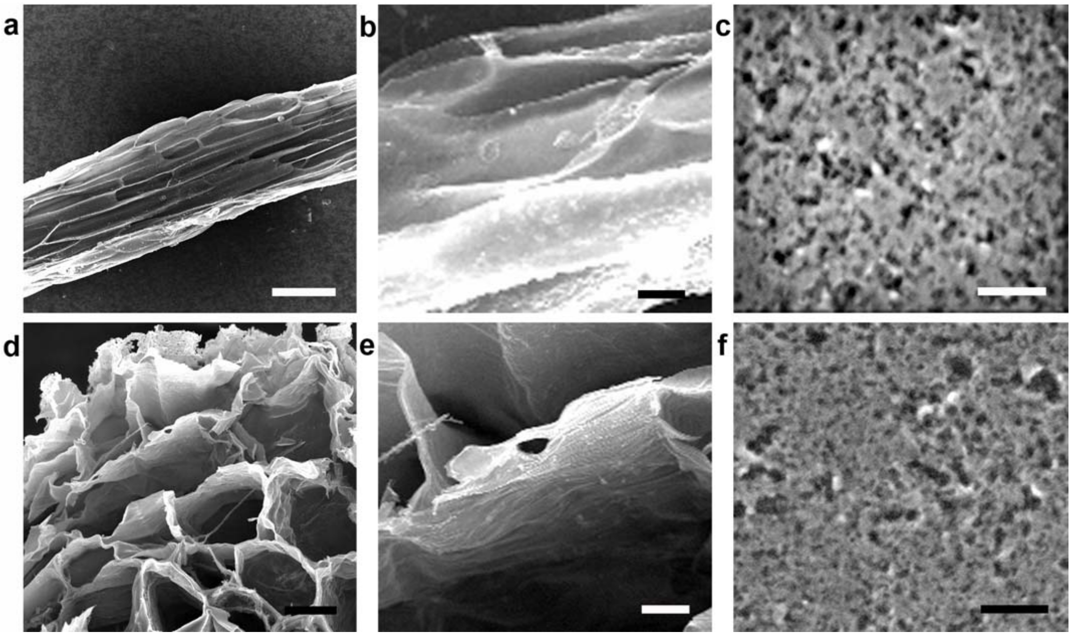

Supplementary Figure 4. Morphology of $3DC/SnO_x$ derived from epidermis of stems ($3DC/SnO_x$-E) and roots ($3DC/SnO_x$-R). (a-c) Longitudinal section scanning electron microscopy (SEM) image of $3DC/SnO_x$-E; (d-f) Cross section of $3DC/SnO_x$-R. The morphology of $3DC/SnO_x$-E and $3DC/SnO_x$-R shows a similar three-dimensional hierarchical porous structure to that of $3DC/SnO_x$ derived from medulla of stems ($3DC/SnO_x$-M). The scale bars in a, b, c, d, e and f are 50 μm, 2 μm, 30 nm, 10 μm, 2 μm and 30 nm.

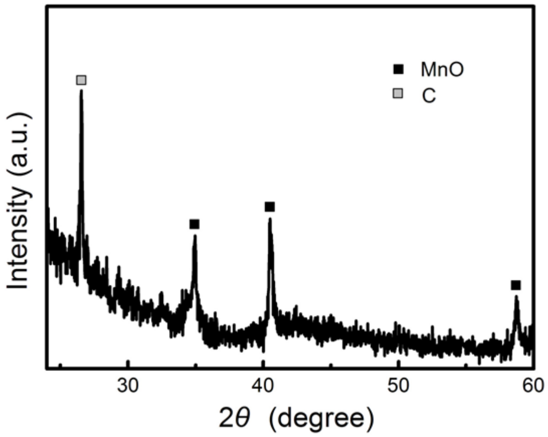

Supplementary Figure 5. X-ray diffraction (XRD) pattern of the 3DC/MnO derived from roots (3DC/MnO-R). In addition to the diffraction peak of 3DC/MnO-R, a strong diffraction peak at 2θ=26.4° corresponding to the interlayer distance of graphitized carbon is observed, suggesting the 3DC is partially graphitized and well ordered.

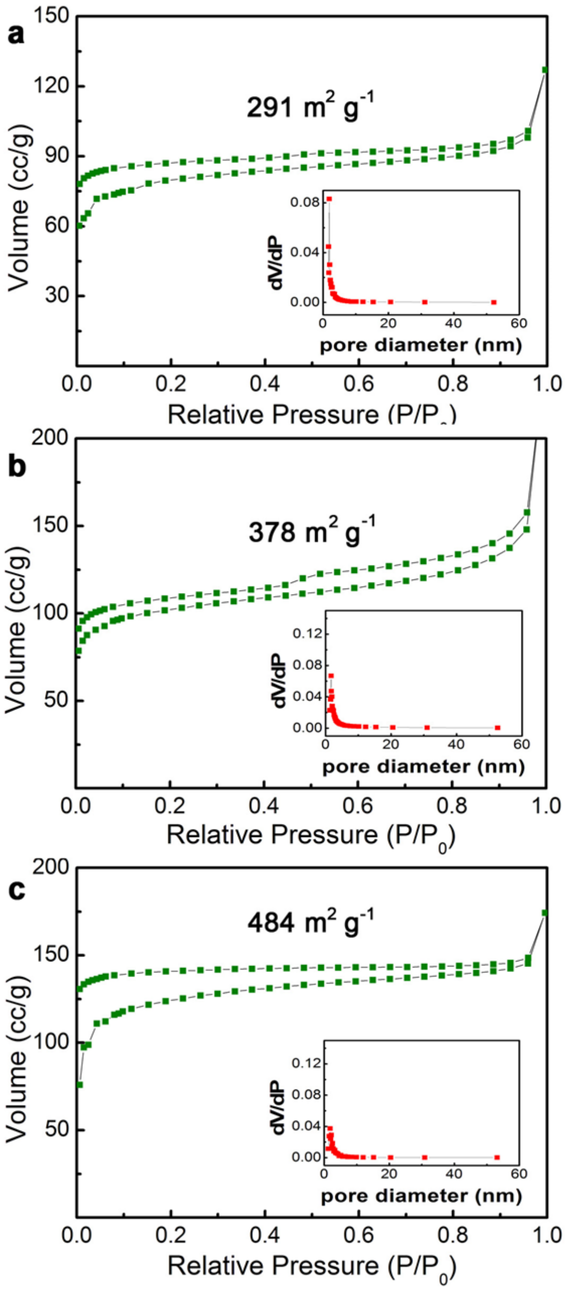

Supplementary Figure 6. Specific surface areas and pore size distributions of 3DC/SnO_x. Nitrogen adsorption/desorption isotherms and the corresponding Barrett Joyner Halenda distributions (inset): (a) 3DC/SnO_x-R; (b) 3DC/SnO_x-E; (c) 3DC/SnO_x-M. All of the three electrode materials display large specific surface area ranged from 291 to 484 m^2 g^{-1}. Additionally, the pores of the 3DC/MO_x are mainly distributed in about 2 nm.

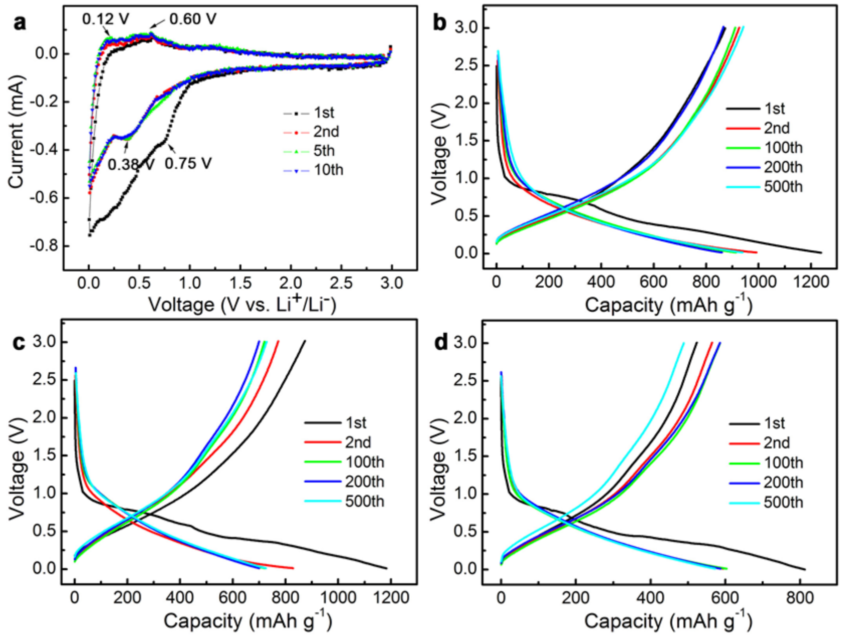

Supplementary Figure 7. Cyclic voltammogram (CV) curves and charge/discharge curves of 3DC/SnO_x. (a) CV curves of 3DC/SnO_x-E at a scan rate of 0.5 mV s^{-1}. The cathodic peak at about 0.75 V in the first cycle, which disappeared in the following cycles, is ascribed to the formation of a solid electrolyte interface (SEI) and the reduction of SnO_x to Sn. Due to the alloying of Li_xSn, a reduction peak started to appear at around 0.38 V from the second cycle. Additionally, two oxidation peaks around 0.12 and 0.60 V stand for the lithium extraction from the 3DC and dealloying of the Li_xSn, respectively. After the initial SEI formation and reduction of SnO_x in the first cycle, both cathodic and anodic peaks show highly stable characteristics, suggesting highly reversible electrochemical cycles. Charge and discharge curves of obtained at a current density of 625 mA g^{-1} in the 1st, 2nd, 100th, 200th, 500th cycles: (b) 3DC/SnO_x-E; (c) 3DC/SnO_x-R; (d) 3DC/SnO_x-M. Overall, the 3DC/SnO_x-E electrode shows a high initial discharge and charge capacity of 1239 and 902 mAh g^{-1} in the first cycle, which reduce to ~888 mAh g^{-1} in about 10 cycle for both the charge and discharge capacity. The 3DC/SnO_x-R and 3DC/SnO_x-M electrodes show a initial discharge capacity of 1183 and 873 mAh g^{-1}, respectively.

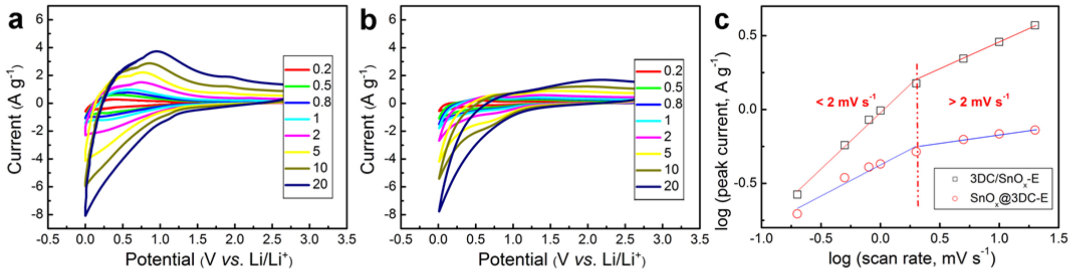

Supplementary Figure 8. CV curves and specific peak current of 3DC/SnO_x-E and 3D carbon derived from epidermis of stems physically/chemically loading SnO_x (SnO_x@3DC-E). (a) CV tests of 3DC/SnO_x-E and (b) SnO_x@3DC-E at increasing sweep rates ranging from 0.2 to 20 mV s^{-1} were performed to investigate the electrochemical behavior in the potential range between 0.01 and 3.0 V (vs Li/Li^+). (c) peak anodic current vs. sweep rate for 3DC/SnO_x-E and SnO_x@3DC-E to evaluate the diffusion characteristics of the electrodes.

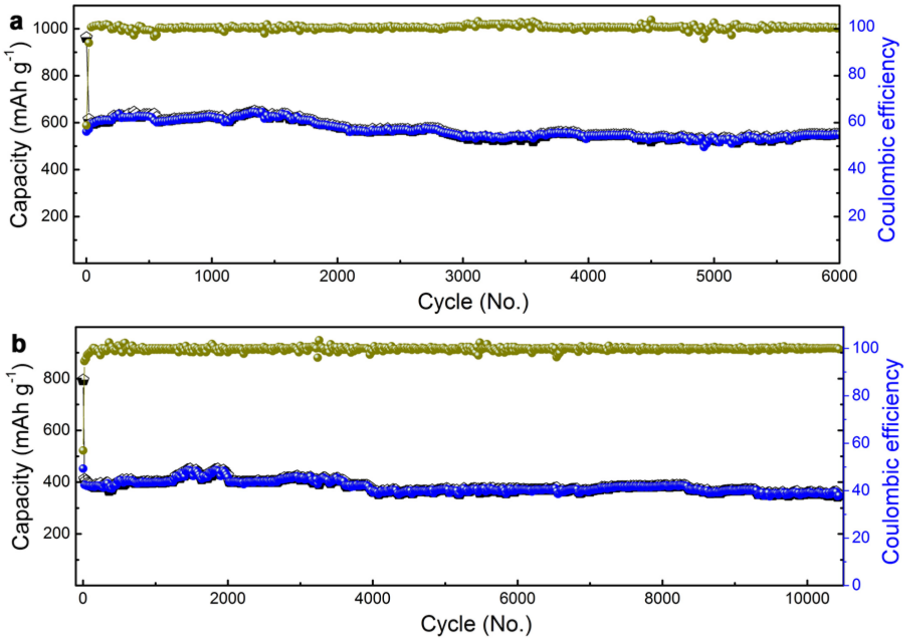

Supplementary Figure 9. Cyclic performance 3DC/SnO_x-E at high current densities. (a) Cyclic performance of 3DC/SnO_x-E at the high current density of 2,500 mA g^{-1}. (b) Cyclic performance of 3DC/SnO_x-E at the high current density of 6,250 mA g^{-1}. The excellent cyclic performance of 3DC/SnO_x-E indicates the 3DC/SnO_x-E could tolerate high current densities with excellent application prospects for high power lithium-ion batteries.

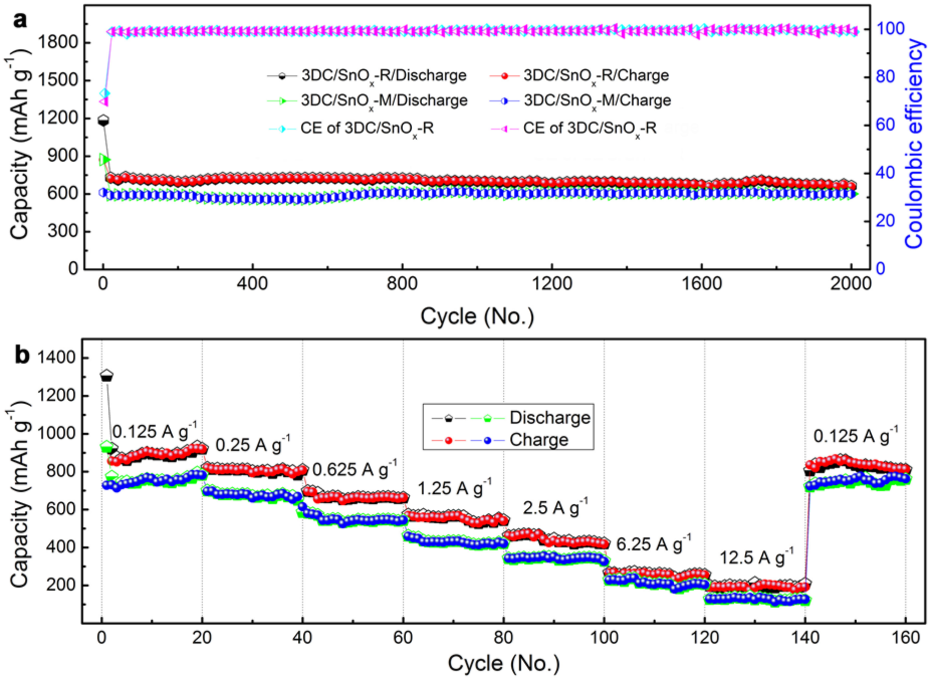

Supplementary Figure 10. Electrochemical performance of 3DC/SnO_x-R and 3DC/SnO_x-M. (a) Cycle performance and Coulombic efficiency (CE) plots of 3DC/SnO_x-R and 3DC/SnO_x-M at a current density of 625 mA g^{-1}. When the current density is delivered at 625 mA g^{-1}, CE of the 3DC/SnO_x-R and 3DC/SnO_x-M rapidly approach 100% after a few cycles and maintain stability, suggesting an excellent cycling stability of the electrodes obtained from roots and medulla of stems. (b) Rate performance of 3DC/SnO_x-R and 3DC/SnO_x-M at the current densities of 125, 250, 625, 1,250, 2,500, 6,250 and 12,500 mA g^{-1}, respectively. Similar to 3DC/SnO_x-E, the 3DC/SnO_x-R and 3DC/SnO_x-M electrodes exhibite excellent rate performance.

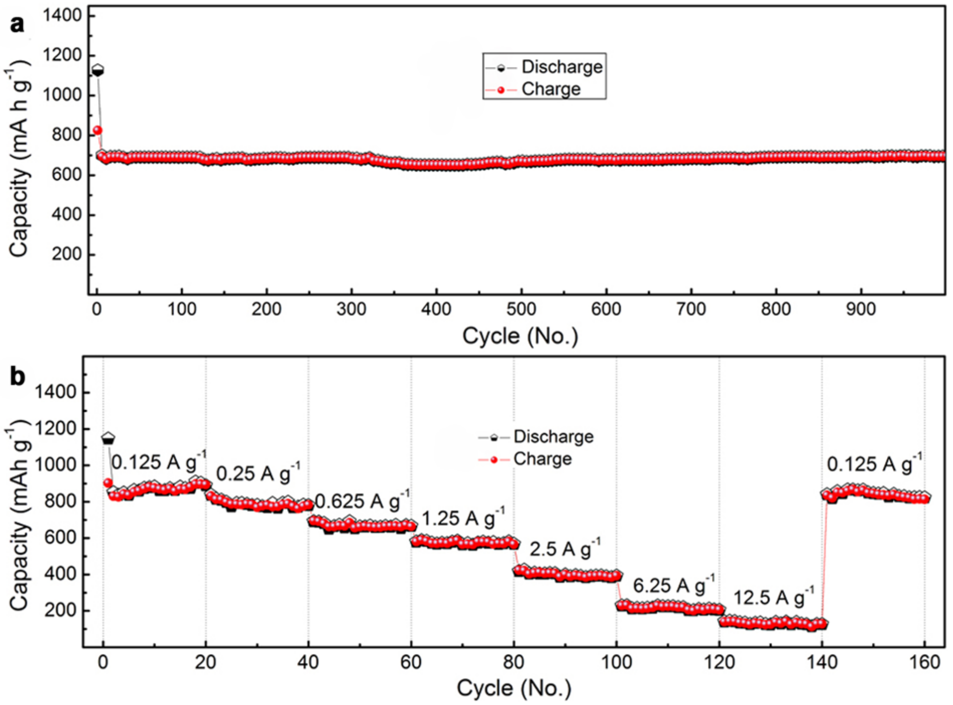

Supplementary Figure 11. Electrochemical performance of the 3DC/MnO-R. (a) Excellent cyclic performance of the 3DC/MnO-R at the current density of 625 mA g^{-1}. Obviously, the 3DC/MnO-R exhibits high reversible capacity and displays discharge capacities of 685 mAh g^{-1} at the current density of 625 mA g^{-1} after 1000 cycles. (b) Capacity versus cycle number plot of the 3DC/MnO-R electrode at various charging rates. The 3DC/MnO-R delivers a specific discharge of 890, 796, 661, 548, 360, 195 and 130 mAh g^{-1} upon increasing the discharge rates to 125, 250, 625, 1,250, 2,500, 6,250 and 12,500 mA g^{-1}, respectively. Moreover, the electrode delivers a specific discharge capacity of about 836 mA h g^{-1} after the current rate returns to 125 mA g^{-1}.

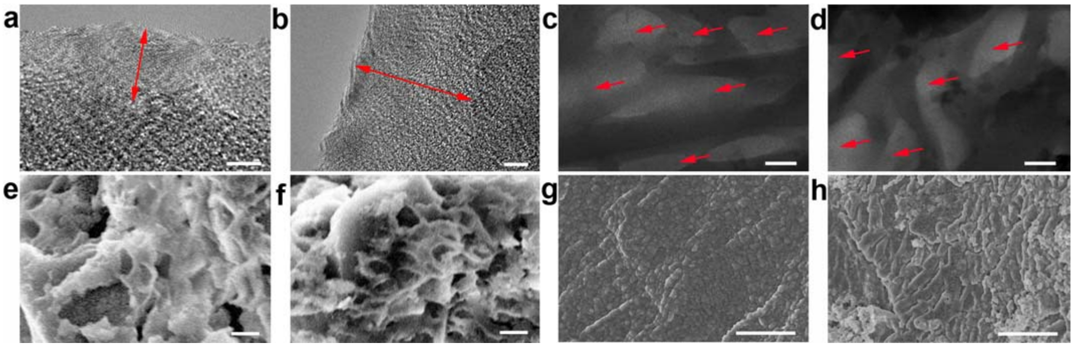

Supplementary Figure 12. Morphology of the 3DC/SnO_x-E electrode after 300 and 3200 cycles. (a, b) High resolution TEM images of the 3DC/SnO_x-E after 300 cycles (a) and 3200 cycles (b), reveal a stable layer of SEI grows from ~10 nm to ~25 nm during the long cycles. (c, d) Low-resolution TEM images of the 3DC/SnO_x-E after 300 (c) and 3200 cycles (d) show the porous architecture of the electrode material is largely intact. (e, f) SEM images of the 3DC/SnO_x-E after 300 (e) and 3200 cycles (f). (g, h) SEM images of the Li foil after 300 (g) and 3200 cycles (h). Morphology of Li foil keep stable after 300 cycles. The SEM images of the Li foil show lithium dendrite growth upon cycling, it shows considerably more mossy surface of Li foil after 3200 cycles compared with that after 300 cycles. The scale bars in a, b, c, d, e, f, g and h are 5, 5, 1, 1, 5, 5, 5 and 5 nm.

Supplementary Table 1. Elemental composition in 3DC/SnO_x-E after washing procedure determined by TEM-EDS.

Element	Si	Cl	N	O	Na	Mg	Al	Ca	Fe	K	Sn	C
Content after washing (wt%)	1.86	0.09	0.84	9.70	0.04	0.14	0.01	0.11	0.03	0.03	40.03	47.12

Supplementary References:

1. Clemens, S.; Palmgren, M. G.; Krämer, U. A long way ahead: understanding and engineering plant metal accumulation. *Trends Plant Sci*. **7**, 309-315 (2002).

2. Gollhofer, J.; Timofeev, R.; Lan. P.; Schmidt. W.; Buckhout, T. J. Vacuolar-iron-transporter1-like proteins mediate iron homeostasis in Arabidopsis. *Plos one* **9**, e110468 (2014).

3. Tester, M.; Leigh, R. A. Partitioning of nutrient transport processes in roots. *J. Exp. Bot*. **52**, 445-457 (2001).

4. Lu, L, *et al*. Efficient xylem transport and phloem remobilization of Zn in the hyperaccumulator plant species Sedum alfredii. *New Phytol*. **198**, 721-731 (2013).
